# Rise of cholera in Iraq: A rising concern

**DOI:** 10.1016/j.amsu.2022.104355

**Published:** 2022-08-18

**Authors:** Khulud Qamar, Umer Usman Malik, Juvairia Yousuf, Mohammad Yasir Essar, Muhammad Muzzamil, Hashim Talib Hashim, Jaffer Shah

**Affiliations:** a Dow Medical College, Karachi, Pakistan; b Afghanistan National Charity Organization for Special Diseases, Kabul, Afghanistan; c Kabul University of Medical Sciences, Kabul, Afghanistan; d Health Services Academy, Islamabad, Pakistan; e University of Baghdad, College of Medicine, Baghdad, Iraq; f Drexel University College of Medicine, Philadelphia, United States

**Keywords:** Cholera, Iraq, Public health, Prevalence, Critical care management

## Abstract

With over seven pandemics and four million reported cases, Cholera remains of the most prevalent acute watery diarrheal diseases in the world to date. As in other developing countries, Iraq once again combats Cholera – and its past encounter in 2015, where the death toll reached 1500, highlights the importance of quickly addressing the current outbreak. The war-torn state of the nation, malnourished public, lack of sanitation and hygiene, mass displacement and global warming all contribute to the prevalence of Cholera in Iraq. Along with the current efforts, additional strategies are recommended for managing cholera cases, such as awareness campaigns, monitoring the safety of water bodies, and food inspection.

## 1 Introduction

The world's struggle against Cholera began during the 19th century in the Ganges delta of India and has since resulted in seven pandemics spanning South Asia, Africa, and the Americas [1]. Our battle with this waterborne bacterial infection is still not over, resulting in an estimate of 1.3–4 million cases and 21,000 to 143,000 deaths globally [1]. Vibrio Cholerae, the causative agent of the disease, is transmitted through a fecal-oral route and, to a lesser extent, by the consumption of shellfish. With over 200 proteins associated with the virulence factor, the serogroups O1 and O139 are the main causes of Cholera, killing individuals of all age groups [2]. When this organism enters the body it travels to the gut lining, where it releases the cholera toxin causing rapid loss of fluid and electrolytes which may progress to severe dehydration, hypovolemic shock and death if left untreated [2]. Symptoms such as watery diarrhea, vomiting, lethargy, and dehydration appear 12 hours to five days after the organism's incubation [1].

The prevalence of Cholera spans many developing countries, owing to poor hygiene and sanitation practices as well as delayed treatment and prevention due to a lack of adequate healthcare infrastructure [3]. As of June 20, 2022, Iraq faces another outbreak of Cholera as the Iraqi Ministry of Health addresses 13 Cholera cases, mainly in the Sulaymaniyah province of the Kurdistan region. Iraq had previously battled Cholera in 2015, where the death toll reached 1500 across 18 provinces, according to the Islamic Relief Project [4].

## 2 Challenges

Iraq has a long history of political instability, socioeconomic crises, and religious tensions which have resulted in shortages of skilled workers, poor availability of resources, and significant healthcare challenges [5]. War tactics have often involved targeted destruction of water infrastructure and distribution systems in order to flood towns, disrupt agriculture, and deprive locals of access to Water, Sanitation, and Hygiene (WASH) resources [6]. Moreover, Iraq's location along the Tigris and Euphrates, whose contaminated waters form the majority of its water supply, and its proximity to neighboring countries with frequent disease outbreaks increases Iraq's vulnerability to waterborne and infectious diseases [5]. Neglect also plays a key role in the crisis: poorly maintained water and sewage systems, lack of proper sewage disposal, open defecation, and a shortage of sanitation engineers have put millions at risk of contracting water-borne diseases, including cholera. Lastly, many citizens cannot afford the high cost of buying water, and instead have to rely on untreated water sources, further leading to poor health outcomes [7].

The people of Iraq are also severely malnourished: food rations provide only half of the daily energy requirement, and inflation has further limited access to food. Furthermore, there is a shortage of medical supplies, equipment, drugs, and skilled personnel. The government has previously shown neglect and has contributed less than the required portion of its budget to healthcare. Hospitals remain underfunded, understaffed, and overcrowded [8].

Although the government has employed methods to prevent the spread of cholera, the overall quality of water and sanitation facilities remains poor. Mass chemoprophylaxis is not preferred due to the risk of developing antimicrobial resistance, and oral cholera vaccines are not recommended in active Cholera outbreaks because of their two-dose schedule, the time required to reach protective efficacy, high cost, and the heavy logistics involved with their use [9]. Moreover, while chlorine for water purification is desperately needed, its provision is often restricted due to its use in past terrorist attacks [10].

Discussion of relief efforts is incomplete without considering the victims of the Islamic State of Iraq and the Levant (ISIL). From 2014 to 2017, ISIL aggression resulted in the displacement of 6 million Iraqi citizens, of which 2.5 million remain in need of acute humanitarian aid due to unsafe living conditions and inadequate healthcare, sanitation, food, and water [11]. Owing to this, a massive cholera outbreak occurred in 2015 as mentioned previously, with cases peaking in October following heavy rainfall [12]. With the role played by these factors in increasing the risk of Cholera well established, strategies aimed at providing aid to the victims of ISIL and the public of Iraq become of paramount importance.

## 3 Efforts and recommendations

Cholera is a self-limiting disease, and timely intervention can reduce mortality by more than 99% [13]. Given the inherent complications of providing healthcare in a post-conflict Iraq, an effective treatment and prevention strategy would employ a multipronged approach involving early detection, management, and treatment of the disease. As of June 2022, the United Nations continues to collaborate with the Ministry of Health of Iraq to improve healthcare outcomes during the Cholera outbreak, and the recent shipment of medical supplies to the Kurdistan region attests to this [14]. At a local level, healthcare authorities remain determined to curtail the spread of the disease and have shown their commitment through the conduction of awareness campaigns [15], monitoring and treatment of water sources [16], and regular food inspections [17]. The promising response shown by the local health authorities remains in line with the United Nations’ goal of having the government take a more proactive approach in providing life-sustaining services to the most vulnerable [18].

Humanitarian organizations continue to work to improve health outcomes amongst Iraqi citizens. For example, the Humanitarian Response Plan (HRP) released by the United Nations Office for the Coordination of Humanitarian Affairs (OCHA) outlined its strategy of aiding 991,000 Iraqis in need, targeting both Internally Displaced Persons (IDPs) and returnees (that is, those that have since returned to their place of origin) [19]. Other measures taken include the construction of a primary healthcare center in Kawergosk, a subdistrict of Erbil, which would cater to the needs of over 20,000 refugees in the region [20]. Lastly, funding from countries, such as Korea [21] and Norway [22] allows for continued humanitarian support, with assistance provided by the United States Agency for International Development (USAID) being especially noteworthy [23].

Sustained efforts resulted in the successful containment of cholera in the outbreaks reported in 2012 [24] and 2015 [25,26]. A 2022 analysis report discussing the achievements made under the HRP plan as set out by OCHA shows that while significant progress was made towards providing healthcare, only 22% of the targeted people have been provided WASH services, with inadequate funding being the predominant challenge [27]. Despite limited successes, the burden of Cholera in Iraq continues to increase beyond expected levels [28].

Cholera is transmitted through the consumption of contaminated food and water, and initial efforts have therefore focused on raising awareness regarding proper handling of such consumables, as well as good hand hygiene [29,30]. WASH programs, through which Iraqi citizens are provided access to water and sanitation services, remain underfunded [27], and therefore priority must be placed on a worldwide effort to provide financial aid to Iraq to ensure continued operation. We suggest that private sanitation facilities, with mirrors and access to liquid soap, be installed as part of WASH efforts, as this may lead to increased adherence to handwashing [31]. Furthermore, while the importance of hand hygiene in curtailing the spread of diseases is well understood, efforts to raise awareness remain ineffective as they fail to consider the determinants that affect handwashing behavior [31,32], and therefore an integrated approach must always be considered when raising awareness regarding hand hygiene; keeping the psychosocial, physical, and environmental needs of people in mind.

In regions where access to clean water remains limited, Oral Cholera Vaccines (OCVs) should be administered, as they have shown to be protective against the disease [33]. WHO pre-qualified OCVs, such as Euvichol-Plus® and Shanchol™ represent vaccines that are both cost-effective [34,35], and easy to store and transport [34,36], and are therefore a promising means of preventing Cholera in countries where it remains endemic, such as in Iraq.

Severe cases of Cholera present with substantial vomiting and diarrhea, and high-risk populations; which include malnourished children, pregnant women, and patients with significant coinfections and comorbidities, require prompt management in clinical settings, where rehydration therapy remains the mainstay treatment [1]. Patients experiencing severe dehydration must be provided with rapid Intravenous (IV) administration of Ringer's Lactate solution corresponding to 10% of their body weight, which can be substituted by normal saline if necessary [37,38]. Initial treatment should readjust for fluid loss caused by ongoing diarrhea to shorten the duration of dehydration, which can be guided by gauging stool volume using Cholera cots [1,37]. During initial therapy, care must be taken to avoid over hydration and subsequent pulmonary edema, especially in pediatric populations [1]. Stabilized patients must be encouraged to use Oral Rehydration Therapy (ORT) when no longer vomiting, and while Glucose-based ORT is widely used, rice-based ORT is more effective in reducing stool volume [39] and is not associated with the production of the Cholera Toxin virulence factor seen with the former formulation [40]. The administration of antibiotics reduces the severity and length of the disease and thus should be co-administered in critical settings in accordance with local antimicrobial resistance patterns; Doxycycline remains the drug of choice due to its single dosage, ease of administration, and cost-effectiveness – even in pregnant women and children [37,41].

In pregnant patients, proper rehydration and maintenance of uterine blood flow is essential for ensuring fetal viability. Furthermore, since the disease can result in the onset of pre-term labor, clinical setups must be well-equipped to deal with such contingencies [1]. Malnutrition is associated with increased mortality, and normal diet should therefore be resumed as soon as possible, while breastfeeding should continue for infants even during the initial treatment [37]. Zinc supplementation of 20 mg/day over a period of ten days is recommended for children aged 5 years and below, and has been shown to reduce both mortality and the risk of future diarrheal illnesses [37].

An effective response to Cholera, however, will remain incomplete without significant upgrades to surveillance systems, which can be used to identify potential outbreaks even before they occur [42]. Data gathered through surveillance systems can aid health authorities in making informed decisions about combatting disease outbreaks, and enable rapid mobilization of medications, rehydration therapies, and vaccines to areas where they are most needed.

Lastly, we would like to emphasize the looming danger of global warming in the context of this disease. The burden of Cholera increases in warmer months [43], as hot temperatures and low levels of precipitation force people to rely on contaminated water resources. We urge that a concerted effort to combat climate change be launched to ensure that the likelihood of Cholera disrupting the lives of thousands of people in developing countries, including Iraq, is kept to a minimum.

## 4 Conclusion

Cholera remains one of the most prominent diseases affecting many parts of the globe, and its causative agent, Vibrio Cholerae, infects individuals of all ages. As Cholera cases once again rise all over the world, Iraq continues its struggle against this disease after its occurrence in 2015. Many factors add to the reason for Cholera's prevalence, including poor infrastructure, lack of efficient healthcare, poor sanitation and hygiene, malnourishment, and the war-torn state of the country. Relief projects continue to be implemented on a large scale through the provision of rehydration therapies, antidiarrheals, vaccinations, food, and financial aid. For long-term improvements in health outcomes, awareness campaigns must be implemented and improvements in infrastructure and public healthcare facilities should be achieved. Further insight and research are required to develop techniques to reduce the incidence of Cholera and decrease its global prevalence.

## Ethical approval

N/A.

## Sources of funding

None.

## Author contribution

All authors equally contributed.

## Consent

N/A.

## Registration of research studies

1. Name of the registry: N/A
2. Unique Identifying number or registration ID: N/A
3. Hyperlink to your specific registration (must be publicly accessible and will be checked): N/A

## Guarantor

N/A.

## Provenance and peer review

Not Commissioned, externally peer reviewed.

## Declaration of competing interest

None declared.

## References

[1] Kanungo S., Azman A.S., Ramamurthy T., Deen J., Dutta S. (2022). https://pubmed.ncbi.nlm.nih.gov/35397865/.

[2] Cho Y.-J., Yi H., Lee J.H., Kim D.W., Chun J. (2010). Genomic evolution of Vibrio cholerae. Curr Opin Microbiol.

[3] Deen J., Mengel M.A., Clemens J.D. (2020). Epidemiology of cholera. Elsevier Ltd.

[4] Cholera Outbreak in Iraq - Islamic Relief Worldwide.

[5] Zolnikov T.R. (2013 Jun). The maladies of water and war: addressing poor water quality in Iraq. Am J Public Health.

[6] Gleick P.H. (2019 Jul). Water as a weapon and casualty of armed conflict: a review of recent water-related violence in Iraq, Syria, and Yemen. WIREs Water.

[7] Iraq: Dirty Water.

[8] https://www.reuters.com/investigates/special-report/iraq-health/.

[9] https://news.un.org/en/story/2007/10/234872-cholera-continues-spread-iraq-un-health-agency-reports.

[10] Iraq https://reliefweb.int/report/iraq/iraq-health-services-struggle-prevent-cholera-spreading.

[11] Iraq O.C.H.A. Humanitarian needs overview 2022. https://reliefweb.int/report/iraq/iraq-humanitarian-needs-overview-2022-march-2022.

[12] https://www.unicef.org/mena/press-releases/iraq-cholera-outbreak-prompts-response.

[13] https://www.who.int/news-room/fact-sheets/detail/cholera.

[14] https://iraq.un.org/en/186938-who-provides-sulaymaniyah-urgent-medical-supplies-prepare-and-respond-recent-cholera.

[15] وزارة الصحة العراقية - Ministry of Health (Iraq) (2022). وزارة الصحة وبالتعاون مع منظمة الصحة العالمية تنفذ ورشة تدريبية لبناء القدرات بشأن المخاطر ومشاركة المجتمع في التوعية [The Ministry of Health, in cooperation with the World Health Organization, implements a training workshop for capacity building on risks [Internet]. https://moh.gov.iq/?page=4124.

[16] وزارة الصحة العراقية - Ministry of Health (Iraq) (2022). توزيع اقراص تعقيم المياه في المناطق غير المخدومة بشبكة الاسالة في قضاء سوق الشيوخ [Distribution of water sterilization tablets in unserved areas of the liquefaction network in Suq Al-Shuyoukh district]. Arabic [Internet]. https://moh.gov.iq/?page=4072.

[17] وزارة الصحة العراقية - Ministry of Health (Iraq) (2022). لليوم الثاني على التوالي صحة كربلاء تنفذ سلسلة من الجولات الرقابية في الاسواق والمحال التجارية في عموم المحافظة [For the second day in a row, Karbala Health is carrying out a series of control tours in markets and shops throughout the governorate]. Arabic. https://moh.gov.iq/?page=4140.

[18] Iraq O.C.H.A. Humanitarian bulletin - overview on humanitarian transition. https://reliefweb.int/report/iraq/iraq-humanitarian-bulletin-overview-humanitarian-transition-may-june-2022.

[19] Iraq O.C.H.A. Humanitarian response plan 2022 - executive summary. https://reliefweb.int/report/iraq/iraq-humanitarian-response-plan-2022-executive-summary-enarku.

[20] Opening U.N.H.C.R. https://reliefweb.int/report/iraq/opening-new-primary-health-care-centre-kawergosk-support-local-and-refugee-communities-enarku.

[21] Un-Habitat (2022). UN-Habitat Iraq receives generous support from Korea International Cooperation Agency (KOICA) to promote peace and stability by facilitating sustainable returns [EN/AR] - Iraq | ReliefWeb [Internet]. https://reliefweb.int/report/iraq/un-habitat-iraq-receives-generous-support-korea-international-cooperation-agency-koica-promote-peace-and-stability-facilitating-sustainable-returns-enar.

[22] UNDP (2022). Norway reaffirms its commitment to stabilization in Iraq with US$ 7.5 million contribution [EN/AR] - Iraq | ReliefWeb [Internet. https://reliefweb.int/report/iraq/norway-reaffirms-its-commitment-stabilization-iraq-us-75-million-contribution-enar.

[23] Unct Iraq W.H.O. (2022). WHO marks five years of strategic partnership with USAID in Iraq - Iraq | ReliefWeb [Internet]. https://reliefweb.int/report/iraq/who-marks-five-years-strategic-partnership-usaid-iraq.

[24] https://reliefweb.int/report/iraq/cholera-iraq-2012.

[25] http://www.emro.who.int/pandemic-epidemic-diseases/news/cholera-cases-decline-in-iraq.html.

[26] UNICEF (2016). https://reliefweb.int/report/iraq/successful-and-timely-efforts-against-killer-disease-are-sustained-2016-enar.

[27] OCHA, Cluster P. Inter-Cluster Analysis of Achievements and Gaps in Implementation of the 2022 HRP | HCT Meeting 5 July 2022 - Iraq | ReliefWeb [Internet]. 2022 [cited 2022 Jul 3]. Available from: https://reliefweb.int/report/iraq/inter-cluster-analysis-achievements-and-gaps-implementation-2022-hrp-hct-meeting-5-july-2022.

[28] Iraq: cholera epidemic - emergency plan of action (EPoA), DREF n° MDRIQ015 - Iraq | ReliefWeb. https://reliefweb.int/report/iraq/iraq-cholera-epidemic-emergency-plan-action-epoa-dref-ndeg-mdriq015.

[29] وزارة الصحة العراقية - Ministry of Health (Iraq) (2022). صحة ديالى تواصل حملاتها التثقيفية حول زيادة الوعي بمرض الكوليرا في بعقوبة [Diyala Health continues its educational campaigns to raise awareness of cholera in Baqubah]. Arabic [Internet. https://moh.gov.iq/?page=4139.

[30] وزارة الصحة العراقية - Ministry of Health (Iraq) (2022). المراكز الصحية لقطاع الگرمة بمحافظة الانبار تكثف حملاتها حول مخاطر الأسهال الوبائي/الكوليرا والحمى النزفية [Al-Garma health centers in Anbar Governorate intensify their campaigns on the dangers of epidemic diarrhea/cholera and hemorrhagic fever]. Arabi [Internet. https://moh.gov.iq/?page=4161.

[31] White S., Heath T., Khalid Ibrahim W., Ihsan D., Blanchet K., Curtis V., Tappis H. (2022). PLoS One [Internet].

[32] White S., Thorseth A.H., Dreibelbis R., Curtis V. (2020). The determinants of handwashing behaviour in domestic settings: an integrative systematic review. International Journal of Hygiene and Environmental Health.

[33] Bi Q., Ferreras E., Pezzoli L., Legros D., Ivers L.C., Date K. (2017 Oct 1). Protection against cholera from killed whole-cell oral cholera vaccines: a systematic review and meta-analysis. Lancet Infect Dis.

[34] Euvichol-Plus® ‘the world's first plastic vial oral cholera vaccine,’ ready for global use – IVI [Internet]. https://www.ivi.int/news-and-stories/press-releases/?mod=document&uid=956.

[35] Ilboudo P.G., Mengel M.A., Gessner B.D., Ngwira B., Cavailler P., Le Gargasson J.B. (2021 Dec 1). https://resource-allocation.biomedcentral.com/articles/10.1186/s12962-021-00270-y.

[36] https://extranet.who.int/pqweb/content/shanchol.

[37] https://medicalguidelines.msf.org/en/viewport/CHOL/english/management-of-a-cholera-epidemic-23444438.html.

[38] https://www.cdc.gov/cholera/treatment/rehydration-therapy.html.

[39] Butler T. (2017). http://academic.oup.com/trstmh/article/111/5/204/4209515/Treatment-of-severe-cholera-a-review-of-strategies.

[40] Kühn J., Finger F., Bertuzzo E., Borgeaud S., Gatto M., Rinaldo A. (2014 Dec 4). https://dx.plos.org/10.1371/journal.pntd.0003347.

[41] https://www.cdc.gov/cholera/treatment/antibiotic-treatment.html.

[42] https://www.who.int/emergencies/surveillance.

[43] Asadgol Z., Mohammadi H., Kermani M., Badirzadeh A., Gholami M. (2019 Nov 1). http://pmc/articles/PMC6834266/.

